# Performance, Fermentation Characteristics and Composition of the Microbiome in the Digest of Piglets Kept on a Feed With Humic Acid-Rich Peat

**DOI:** 10.3389/fvets.2019.00029

**Published:** 2019-02-12

**Authors:** Christian Visscher, Julia Hankel, Andrea Nies, Birgit Keller, Eric Galvez, Till Strowig, Christoph Keller, Gerhard Breves

**Affiliations:** ^1^Institute for Animal Nutrition, University of Veterinary Medicine Hannover, Foundation, Hannover, Germany; ^2^Helmholtz Center for Infection Research, Braunschweig, Germany; ^3^Boehringer Ingelheim Veterinary Research Center GmbH & Co. KG, Hannover, Germany; ^4^Institute for Physiology, University of Veterinary Medicine Hannover, Foundation, Hannover, Germany

**Keywords:** fermentation characteristics, humic acid, microbiome, peat, performance

## Abstract

The transition from breast milk to solid feed is a dramatic change in the nutrition of piglets, frequently necessitating antibiotic treatment. In efforts to reduce the use of antibiotics, dietetic concepts based on natural feed additives are becoming more and more important. In the present study, experiments were carried out with 15 rearing piglets (days 28–56) divided into three groups that were offered different diets (Ctr [0% peat]; H1.5 [1.5% peat]; and H3.0 [3.0% peat] based on a commercial weaner recipe; all ~178 g CP, 13.7 MJ ME, 13.3 g Lys, as-fed). The contents of cecal and colon digesta were removed at necropsy. The gas formation (4 h) in colon digesta was measured using *in vitro* batch fermenters. For microbiome studies, 16S rRNA amplification was performed within the hypervariable region V 4 and sequenced with Illumina MiSeq platform. DNA read mapping and statistical analysis were performed using QIIME (version 1.8.0), MicrobiomeAnalyst, RStudio, and SAS Enterprise Guide. The mean body weight of the animals at the end of the trial did not show statistical differences (in kg: Ctr: 26.1 ± 4.85, H1.5: 28.5 ± 3.41, H3.0: 26.2 ± 4.92). The daily weight gains were high for this age (in g/day; Ctr: 607 ± 157; H1.5: 692 ± 101; H3.0: 615 ± 113) and the feed to gain ratio low (in kg/kg; Ctr: 1.538; H1.5: 1.462; H3.0: 1.462). Concentrations of short-chain fatty acids in the cecal content were significantly lower when peat was used (mmol/kg wet weight; Ctr: 173 ± 30.0; H1.5:134 ± 15.0; H3.0:133 ± 17.3). Numerical differences were found in the gas formation (in mL gas per 10 mL batch in 4 h; Ctr: 7.9 ± 2.2; H1.5: 7.4 ± 2.4; H3.0: 6.6 ± 1.1). The microbiome analyses in the cecal content showed significantly higher values for alpha diversity Chao 1 index for samples from the control group. Significant differences were found for bacterial relative abundance for *Tenericutes* at phylum level and *Mollicutes* at class level (*p* < 0.05) in cecal microbiota. Therefore, there was initial evidence that peat influences intestinal microflora causing a shift in the overall concentration of fermentation products in both, the cecal and the colon content.

## Introduction

Weaning imposes tremendous stress on piglets (1, 2). The piglets have to cope with the sudden withdrawal of sow milk and adapt to less digestible, plant-based dry diets containing complex protein, and carbohydrate including various anti-nutritional factors (1). Marked changes in fermentation activities and microbial ecology may occur when altering the diet (2). Access of pathogens to the disturbed ecosystem is alleviated (2). Therefore, the period following weaning can be characterized by a high incidence of intestinal disturbances with diarrhea and depression of growth performance in piglets (1, 2), causing significant economic losses in pig farming (3).

Extensive research on the specific use of feed ingredients and feed additives has been undertaken to reduce the industry's dependence on current antimicrobial compounds for controlling problems associated with the weaning transition without using antimicrobial compounds (1, 4–6).

As one of the alternative feed additives, humic substances (including humates, humifulvates, humic acids, and fulvic acid) have been used in animal husbandry to improve the economics and ecology of animal production by increasing the growth rate, improving feed efficiency and immunity, and reducing the risk of disease (7, 8). However, dietary supplementation with humic substances in pig feed has not yet been fully investigated (9, 10). In recent years, interest in the use of humic substances or rather peat as a feed additive has increased, in particular because of its ability to prevent intestinal diseases and stimulate the growth of piglets and pigs (11, 12). If positive effects were seen, these were most likely associated with a high content of humic acids and other organic and inorganic substances (11). Humic substances are defined as “a series of relatively high-molecular-weight, yellow to black colored substances formed by secondary synthesis reactions” (13). Dietary humic acids have been shown to increase the average daily weight gains and feed to gain ratio of young pigs (14, 15) and sometimes to exert no influence (12, 16).

Humic substances may alter microbiota in the intestinal digesta (17, 18). Some recent studies on ruminants do in fact show that humic acid could be used to modulate the ruminal fermentation pattern by shifting ruminal fermentability to more efficient end products (17). Humic substances decreased the relative abundance of *Proteobacteria* (*p* = 0.04) and increased the relative abundance of *Synergistetes* (*p* = 0.01) and *Euryarchaeota* (*p* = 0.04) (18). In studies on humic substances in pigs, correlation analysis in the control (without humic substances) in general showed a positive correlation of the ETEC-infected control with the genera *Turicibacter, Clostridium, Campylobacter, Dehalobacterium, Desulfuvibrio*, and *Paludibacter* and a negative correlation with the genera *Prevotella, Blautia, Faecalibacterium, Lactobacillus*, and *Coprococcus* (19). Inverse correlations with these genera were observed in the supplemented groups, especially in the sodium humate + ZnO group. The results indicate that dietary supplementation with sodium humate + ZnO affects the microbial composition of feces while maintaining good health condition and growth performance in ETEC-infected weaned pigs (19).

The present study hypothesized that the use of peat influenced the microbiota in the gastrointestinal tract of young pigs, which should be reflected in corresponding parameters (fermentation products, gas production *in vitro* etc.) without the performance of the animals deviating from a normal level.

## Materials and Methods

Animal experiments were performed in accordance with German regulations. These animal experiments require no notification or approval in accordance with the Animal Protection Act (§7, paragraph 2, sentence 3). Interventions before dissection were not carried out. The animals were killed in accordance with §4, paragraph 3 of the Animal Protection Act, exclusively to use their organs or tissues for scientific purposes. The experiments were approved by the Animal Welfare Officer of the University of Veterinary Medicine Hannover, Germany (reference: “Tötungsanzeige beim Tierschutzbeauftragten der Stiftung Tierärztliche Hochschule Hannover”, 29.01.2018).

### Animals and Housing

Fifteen crossbred piglets (Topigs Norsvin, Senden Deutschland; TN70 x Pietrain) from a commercial pig farming were used. The piglets had been vaccinated against *Mycoplasma hyopneumoniae* (Suvaxyn MH One, Zoetis Deutschland GmbH, Berlin, Germany) and the *Escherichia coli* shigatoxin 2e antigen (Ecoporc Shiga, IDT Biologika GmbH, Dessau-Roßlau, Germany). Piglets were weaned on day 21 of life. Subsequently, the piglets were transported directly to the University of Veterinary Medicine Hannover, Foundation. The animals were housed in three identically equipped boxes on concrete floors with wood shavings as bedding (GOLDSPAN®, Goldspan GmbH, and Co. KG, Goldenstedt, Germany) in accordance with regulations on the protection of animals and the keeping of production animals. The bedding was renewed regularly.

### Feeding Concept, Experimental Design, and Necropsy

The animals had free access to water and feed. The feeding was based on using complete feedingstuffs. In an adaptation phase of about 1 week, the change to the respective experimental diets took place. The feeding trial itself lasted for 4 weeks (days 28–56 of life). For the trial, three different granulated diets were produced in accordance with the recommended requirements for rearing piglets in Germany (20) (Rothkötter Mischfutterwerk GmbH, Meppen Versen, Germany) consisting of wheat, barley, soybean meal, wheat bran, oat flour, confectionary products, wheat flour, macerated wheat, beet pulp, sunflower seed extract, calcareous lime, fish concentrate, plant oil, monocalciumphosphate, sodium chloride, plant fatty acid, fish oil, sodium bicarbonate, and feed additives (Table 1) in descending order. The control diet contained zero percent peat (Ctr), the respective experimental contained 1.5% peat (Futtergold, Deutsche Torf-Gesellschaft mbH, Scharrel, Germany; H1.5) or 3.0% peat (H3.0). Diets were isoenergetic and isonitrogen and balanced with regard to the essential amino acids content (Table 1). During the experimental phase, performance parameters were recorded, i.e., feed intake and weights were recorded on a weekly basis. Corresponding parameters (average daily weight gain [ADWG] and feed conversion ratio [FCR]) were determined. On the day of necropsy, the animals had *ad libitum* access to water and feed before being removed from the group. Stunning took place by penetrating bolt shot in accordance with Article 4(1) in conjunction with Annex I of the Council Regulation (EC) No 1099/2009. Subsequently, animals were killed by blood withdrawal. At necropsy, samples from cecal and colon content were collected for use in further investigations.

**Table 1:** Concentrations of ingredients and energy content after chemical analysis in control and experimental diets (88% DM).

**Item**		**Ctr**	**H1.5^a^**	**H3.0^b^**
Crude ash	[g/kg]	47.2	46.6	45.8
Crude fat		38.0	41.8	45.4
Crude fiber		35.4	35.3	35.3
Crude protein		180	176	177
Starch		402	407	403
Metabolisable energy	[MJ/kg]	13.7	13.7	13.8
Calcium [g/kg]	[g/kg]	6.13	6.14	6.35
Phosphorus		4.95	4.74	4.68
Copper	[mg/kg]	151	158	165
Iron		505	453	468
Selenium		0.36	0.36	0.33
Zinc		130	128	128
Lysine	[g/kg]	13.4	13.3	13.3
Methionine		3.61	3.83	3.47
Acid detergent fiber		47.4	52.1	58.5
Neutral detergent fiber		140	140	132

*Ctr [0% peat], H1.5 [1.5% peat], and H3.0 [3.0% peat]; additives [per kg as fed]; nutritional additives: vitamin A (10,000 IU), vitamin D/vitamin D3 (2,000 IU), vitamin E (80 mg), iron from iron-(II)-sulfate monohydrate (100 mg), copper from copper-(II)-sulfate pentahydrate (160 mg), manganese from manganese-(II)-sulfate (80 mg), zinc from zinc sulfate monohydrate (100 mg), iodine from calcium iodate anhydrous (2.0 mg), selenium from sodium selenite (0.35 mg); zootechnical additives: 6-phytase EC 3.1.3.26 (1,000 phytase activity units [FTU]), endo-1,4-beta-xylanase EC 3.2.1.8 (200 FYT), benzoic acid (5000 mg); technological additives: formic acid, calcium formate, montmorillonite (1,000 mg)*.

a H1.5 was a mixture of 50% Ctr and 50% H3.0 diet

b *The humic acid analysis was carried out only in the H3.0 diet. This diet contained 1.06% extractable humic acids*.

### Analyses

#### Chemical Analyses

All diets were analyzed in accordance with standard methods of the VDLUFA (21). The dry matter content was determined by drying to a constant weight at 103°C. The crude ash content was analyzed by combustion in a muffle furnace at 600°C for 6 h. The total nitrogen content was determined using the DUMAS combustion method (Vario Max, Elementar, Hanau, Germany). The crude fat content was determined in accordance with the standard protocol in the Soxhlet apparatus. The crude fiber was analyzed after washing in diluted acids and alkalis. Starch determination was carried out polarimetrically (Polatronic E, Schmidt und Haensch GmbH & Co., Berlin, Germany). The minerals were analyzed by atomic absorption spectrometry (Unicam Solaar 116, Thermo, Dreieich, Germany). The amino acids were determined by ion-exchange chromatography (AA analyser LC 3000, Biotronic, Maintal, Germany). The volatile fatty acids content in the homogenized cecal chyme was measured with a gas chromatograph (610 Series, Unicam, Kassel, Germany). After mixing the sample with the internal standard (10 mL of formic acid [89%] and 0.1 mL of 4-methylvaleric acid), the mixture was centrifuged and then subjected to gas chromatography with a column temperature of 155°C (injector: 175°C, detector: 180°C). The humic acid analyses were carried out in accordance with internal, constantly revised, and tested analysis regulations and procedures of the iTN-Zittau, c/o Hochschule Zittau/Görlitz, Germany in accordance with published procedures (22).

#### Batch Fermentation

**Figure 1:**
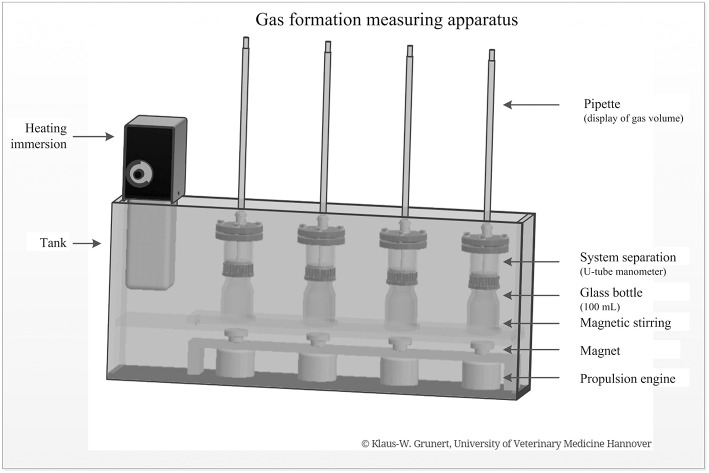
Gas formation measuring apparatus with single components for batch fermentation of intestinal chyme (developed and manufactured by Klaus-W. Grunert, University of Veterinary Medicine, Foundation, Hannover).

A specially developed incubation apparatus was used for batch fermentation (Figure 1). The device consisted of a heatable and temperable tank. The heating immersion unit was used to set a physiological temperature range (body temperature). Each tank contained several measuring units consisting of a glass bottle (250 mL, Schott Duran®) with a magnetic stirring and an attached system separation ending with a graduated pipette. Below the glass bottle, there was a magnet and a propulsion engine. For batch fermentation, the contents of the colon were filtered through several layers of gauze, separated according to the feeding groups, and evenly distributed on reaction fermenters. In a subsequent step, 60 mL liquid chyme and 27 mL starch solution were incubated (4.44 g starch powder dissolved in 100 mL physiological isotonic buffer solution). This was done in a water bath at 40°C in each case. Stirred magnets ensured complete mixing of the respective preparations. The gas production of the chymus preparations was measured and read using a graduated pipette (cumulatively in mL/4 h). These values were documented at intervals of 5 min over 4 h. For each animal, the batch fermentations runs were examined in duplicate or triplicate.

#### Microbiome Analyses

Samples were stored at −80°C until chyme of each individual animal was homogenized in a mixer mill (Retsch MM 400, Haan, Germany) for 1 min. DNA-extraction was done on an automated liquid handler (Microlab Star, Hamilton Germany GmbH, Planegg, Germany) based on the DNeasy Blood&Tissue Kit (Qiagen, Hilden, Germany) from individual samples. An additional purification step (Kit: BS 365, BioBasic, Ontario, Canada) was performed before Illumina MiSeq 250 bp paired-end sequencing of the hypervariable region V4 was employed for individual samples (*n* = 5 for group and sample location each; in total: *N* = 30). Further steps of the Protocol followed the methodology already described (23).

### Statistical Analyses

The statistical evaluation was carried out using a statistical analysis system software for Windows, the SAS® Enterprise Guide®, Version 7.1 (SAS Institute Inc. Cary, USA). Model residuals were tested for normal distribution using the Shapiro-Wilk test and Kolmogorow-Smirnov test as well as visual evaluation of the Q-Q plots. Since the data could be regarded as normally distributed except for some, a comparison of the three groups was carried out using one-way ANOVA (Fisher's Least-Significant Difference; LSD). DNA read mapping and statistical analysis were performed using QIIME (version 1.8.0), MicrobiomeAnalyst, and RStudio (version 3.5.0) with the phyloseq package (version 1.26.0). Only operational taxonomic units (OTUs) occurring at a relative abundance ≥ 0.2% total reads in at least one sample were kept and further analyzed in RStudio. ANOVA was used to compare univariate analysis of alpha diversity measures. Probability of error α was fixed at 5%.

## Results

### Performance Parameters

The study showed no differences in performance parameter between groups (Table 2). At the beginning of the study, the group mean values deviated from the total mean by a maximum of 0.8% (mean: 8.91 kg; Ctr: 100.2%; H1.5: 100.6%; H3.0: 99.2%), whereas at the end of the study the deviations of the respective group from the mean of all animals were numerically greater (mean: 26.93 kg; Ctr: 96.92%, H1.5: 105.8%, H3.0: 97.29%). Overall, the average daily body weight gains (mean: 638 g/day; Ctr: 95.14%, H1.5: 108.5%, H3.0: 96.39%) were high for this age and the feed conversion ratio excellent (mean: 1.487 kg feed/kg weight gain; Ctr: 103.4%, H1.5: 98.30%, H3.0: 98.30%).

**Table 2:** Performance data of piglets depending on feeding concept.

**Item**		**Period**	**Ctr**		**H1.5^a^**		**H3.0**	
			**Mean**	***SD***	**Mean**	***SD***	**Mean**	***SD***
Body weight	[kg]	Start (age: 28 days)	8.93	0.92	8.96	0.54	8.84	1.70
		Final (age: 56 days)	26.1	4.85	28.5	3.41	26.2	4.92
ADWG	[g/day]	Start-final	607	157	692	101	615	113
FCR	[kg/kg]	Start-final	1.538		1.462		1.462	

*Ctr [0% peat], H1.5 [1.5% peat], and H3.0 [3.0% peat]*;

a *H1.5 was a mixture of 50% Ctr and 50% H3.0 diet*.

### Characterization of the Intestinal Chyme

The nitrogen content (shown as calculated crude protein content, factor 6.25xN; Table 3) in the cecal content of the Ctr group was significantly higher when adding 1.5% peat (H1.5). Animals fed 3.0% peat in their diet (H3.0) showed no difference in the nitrogen content in cecal chyme (dry matter content [DM]; mean: 154 g/kg DM; Ctr: 108.0%; H1.5: 95.0%; H3.0: 97.0%) to either of the other two groups. There were no differences either in the cecal content or in the colon content with regard to the other investigated parameters (DM, starch, Cu, Fe, and Zn).

**Table 3:** Characterization of the cecal and colon content concerning dry matter, starch, crude protein, and trace element contents.

**Sample type**	**Parameter**		**Ctr**		**H1.5^1^**		**H3.0**	
			**Mean**	***SD***	**Mean**	***SD***	**Mean**	***SD***
Cecal content	Dry matter (DM)	[g/kg fresh matter]	123	14.0	119	5.96	126	15.1
	Starch	[g/kg DM]	52.4	8.59	51.8	9.85	51.9	9.56
	Crude protein		166^a^	13.6	146^b^	14.8	149^ab^	10.2
	Cu	[mg/kg DM]	590	81.1	578	68.8	524	74.1
	Fe		1294	174	1226	208	1453	789
	Zn		428	79.7	363	72.3	374	51.9
Colon content	Dry matter	[g/kg fresh matter]	173	14.0	172	19.5	171	25.9
	Starch	[g/kg DM]	46.5	7.28	42.2	5.15	40.3	9.66
	Crude protein		203	14.9	190	18.2	188	21.7
	Cu	[mg/kg DM]	800	81.9	831	96.2	780	105
	Fe		1698	241	1680	197	1983	1331
	Zn		634	62.2	592	80.1	518	114

*Ctr [0% peat], H1.5 [1.5% peat], and H3.0 [3.0% peat]*;

1 *H1.5 was a mixture of 50% Ctr and 50% H3.0 diet*;

a, b *values within a row with different superscripts differ significantly at p < 0.05*.

The use of peat changed the fermentation pattern in the cecal and colon contents (Table 4). In the cecal content of the control animals, the concentration of acetic acid was significantly highest, whereas the concentration of propionic acid in the cecal content and acetic and propionic acid in the colon content differed only significantly between Ctr and H3.0. The sum of short-chain fatty acids (SCFA) in cecal content was highest in the control group (mean: 146.7 mmol/kg fresh matter; Ctr: 117.9%, H1.5: 91.34%, H3.0: 90.66%); in the colon content, this was highest only compared to group H3.0 (mean: 154.0 mmol/kg fresh matter; Ctr: 108.4%, H1.5: 103.9%, H3.0: 87.66%).

**Table 4:** Characterization of the fermentation products in the cecal and colon contents.

**Sample type**	**Parameter**		**Ctr**		**H1.5^1^**		**H3.0**	
			**Mean**	**SD**	**Mean**	**SD**	**Mean**	**SD**
Cecal content	Acetic acid	[mmol/kg fresh matter]	97.6^a^	14.1	77.6^b^	8.20	76.2^b^	3.17
	propionic acid		48.7^a^	8.87	37.8^ab^	5.00	33.8^b^	13.4
	i-butyric acid		0.26	0.11	0.16	0.20	0.20	0.12
	n-butyric acid		24.2	9.06	16.9	5.44	20.2	4.31
	i-valeric acid		0.43	0.14	0.32	0.28	0.33	0.18
	n-valeric acid		1.49	1.08	1.01	0.85	2.05	2.50
	caproic acid		0.17	0.14	0.10	0.09	0.11	0.05
	Total SCFA		173^a^	30.0	134^b^	15.0	133^b^	17.3
Colon content	Acetic acid	[mmol/kg fresh matter]	90.7^a^	10.1	88.0^ab^	9.35	77.7^b^	8.27
	Propionic acid		43.8^a^	9.23	42.5^ab^	5.08	32.6^b^	7.77
	i-butyric acid		1.01	0.35	1.10	0.72	0.73	0.35
	n-butyric acid		26.6	5.73	24.3	6.00	20.7	3.51
	i-valeric acid		1.34	0.50	1.52	0.88	1.00	0.51
	n-valeric acid		2.92	1.52	2.64	1.47	2.71	2.46
	Caproic acid		0.16	0.19	0.21	0.11	0.08	0.07
	Total SCFA		167^a^	23.7	160^ab^	19.1	135^b^	11.4

*Ctr [0% peat], H1.5 [1.5% peat], and H3.0 [3.0% peat]; SCFA, short chain fatty acids*;

1 *H1.5 was a mixture of 50% Ctr and 50% H3.0 diet*;

a, b *values within a row with different superscripts differ significantly at p < 0.05*.

### Batch Fermentation

There was no statistical difference in gas formation for the respective dietetic concepts (Table 5). All incubation approaches were characterized by an initially strong increase in gas production with the cumulative gas formation proceeded in the sense of a saturation curve reaching a plateau area after about 3 h (data not shown).

**Table 5:** Gas formation [mL/4 h] in colon chymus *in vitro* using the colon simulation technique (Cositec).

**Run**	**Ctr**			**H1.5^a^**			**H3.0**		
	**Repetition**	**Repetition**	**Repetition**						
	1	2	3	1	2	3	1	2	3
1	10.3	9.9	–	8.0	8.3	11.3	6.6	6.1	9.3
2	8.2	7.7	–	8.7	8.4	8.1	7.0	7.1	–
3	5.6	5.6	5.9	4.9	4.6	4.7	5.1	5.6	–
Mean ± SD^b^	7.9 ± 2.2			7.4 ± 2.4			6.6 ± 1.1		

*Ctr [0% peat], H1.5 [1.5% peat], and H3.0 [3.0% peat]*;

a *H1.5 was a mixture of 50% Ctr and 50% H3.0 diet*;

b *A statistical evaluation was carried out using one-way ANOVA. Due to the very small sample size, a normal distribution was assumed*.

### Intestinal Microbiome

The results of the microbiome analyses were evaluated separately for the localizations cecum and colon.

#### Alpha Diversity

**Figure 2:**
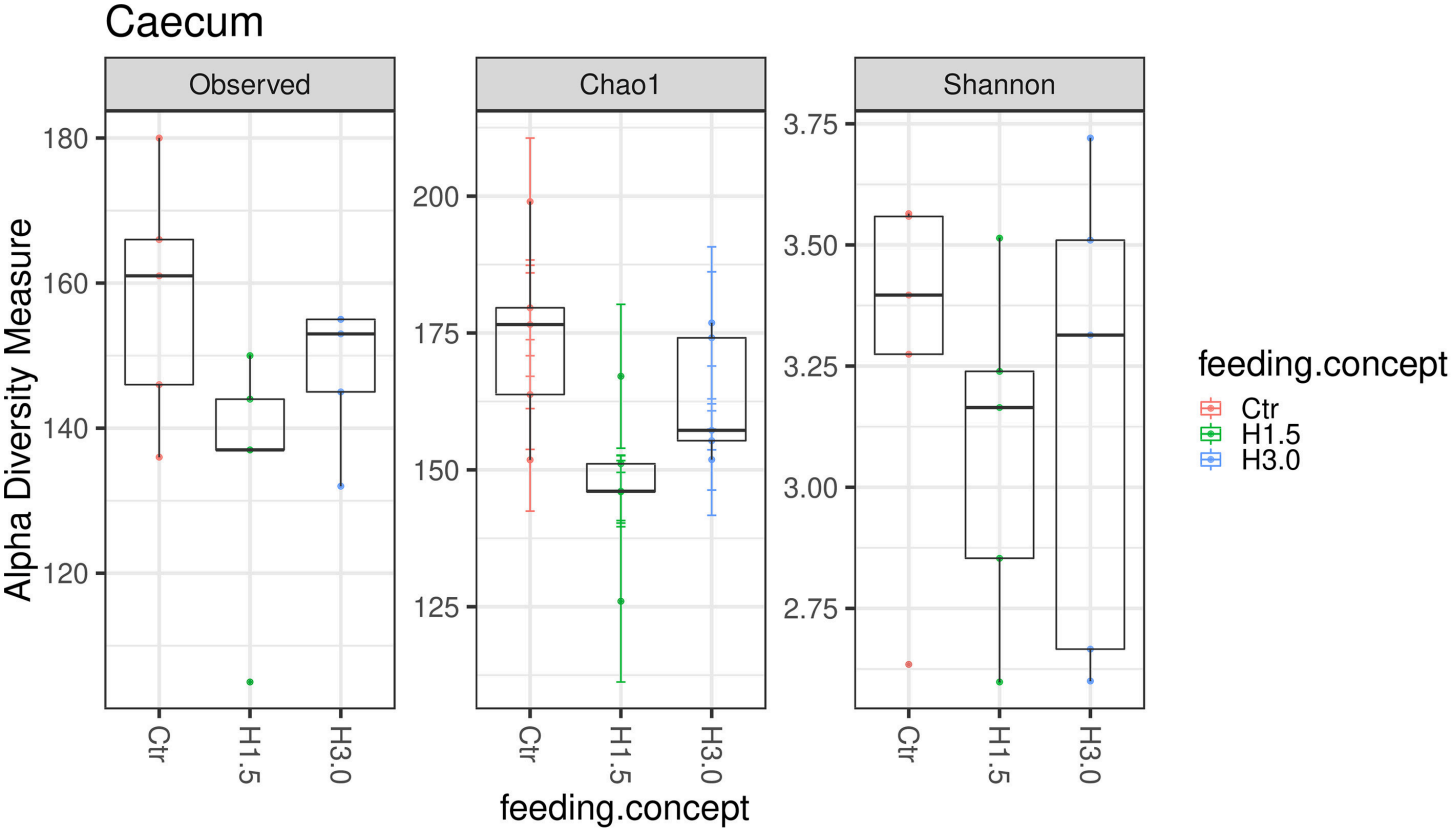
Box-plots showing alpha diversity in samples using the Observed species index, the Chao1 index, and Shannon index in samples from cecal content depending on the feeding concept (Ctr [0% peat]; H1.5 [1.5% peat], and H3.0 [3.0% peat].

The microbiome analyses in the cecal content showed significantly higher values for the Chao 1 index (microbial richness, *p* = 0.0432) for samples from the control group (Table 6, Figure 2). Neither the Observed species index (microbial richness, *p* > 0.05), nor the Shannon index (diversity, *p* > 0.05) differed between groups.

**Table 6:** Alpha-diversity in the microflora of the cecal and colon content (Observed species, Chao1, and Shannon indices) related to feeding concept.

**Sample type**	**Statistic index**	**Ctr**		**H1.5^a^**		**H3.0**		***P*-value**
		**Mean**	***SD***	**Mean**	***SD***	**Mean**	***SD***	
Cecal content	Observed	158	17.2	135	17.4	148	9.85	0.0925
	Chao 1	174	17.7	147	14.7	163	11.5	0.0432
	Shannon	3.28	0.384	3.07	0.355	3.16	0.504	0.731
Colon content	Observed	166	15.4	145	12.3	156	16.2	0.119
	Chao 1	173	14.6	157	19.2	166	16.9	0.361
	Shannon	3.39	0.387	3.27	0.189	3.32	0.183	0.771

*Ctr [0% peat], H1.5 [1.5% peat], and H3.0 [3.0% peat]*;

a *H1.5 was a mixture of 50% Ctr and 50% H3.0 diet*.

**Figure 3:**
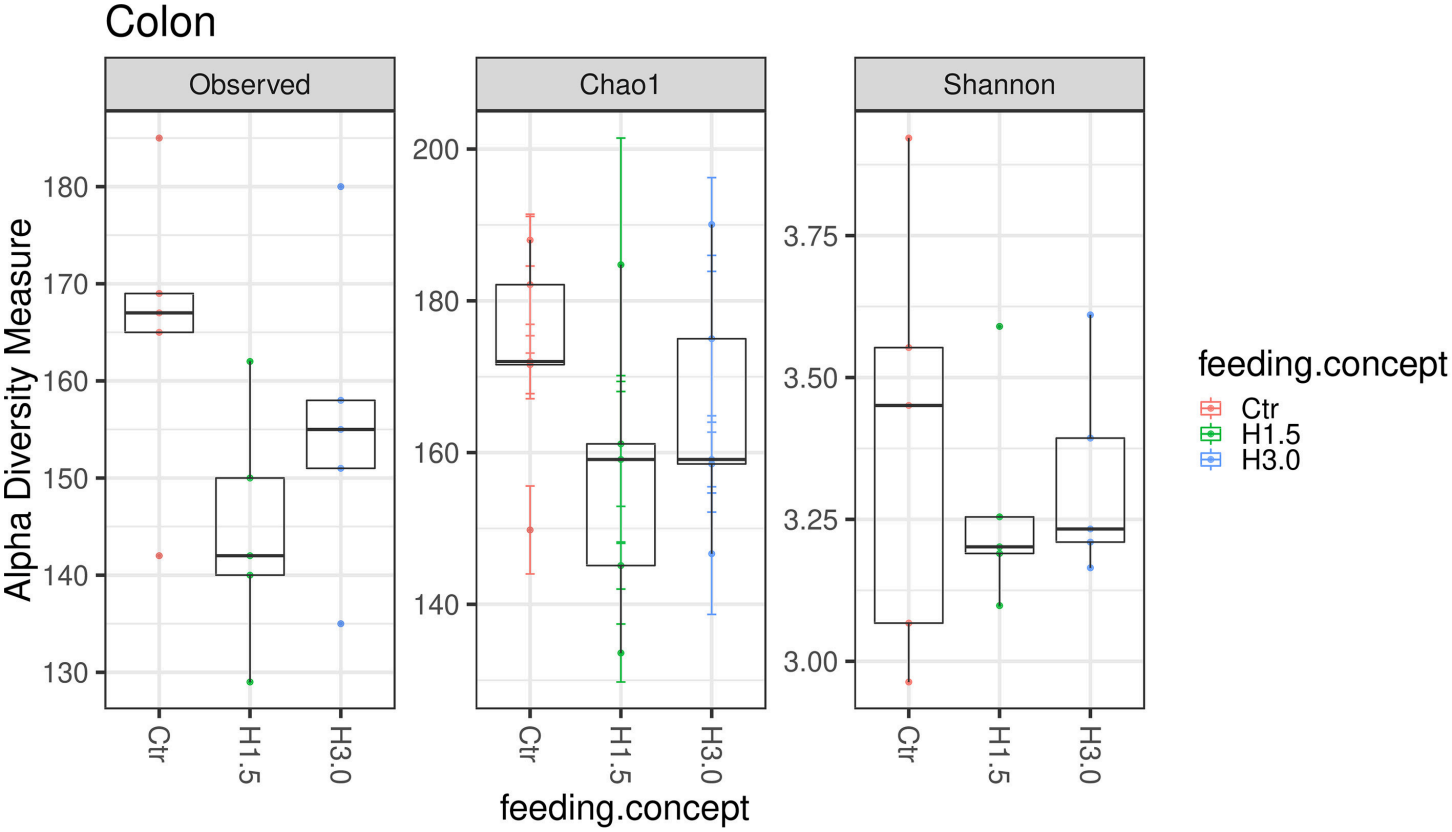
Box-plots showing alpha diversity in samples using the Observed species index, the Chao1 index, and Shannon index in samples from colon content depending on the feeding concept (Ctr [0% peat]; H1.5 [1.5% peat], and H3.0 [3.0% peat].

In the colon content, no significant differences could be observed in the microbial analysis regarding the Observed species index (microbial richness, *p* > 0.05), the Chao 1 index (microbial richness, *p* > 0.05) and the Shannon index (diversity, *p* > 0.05, Figure 3).

#### Relative Abundance

**Figure 4:**
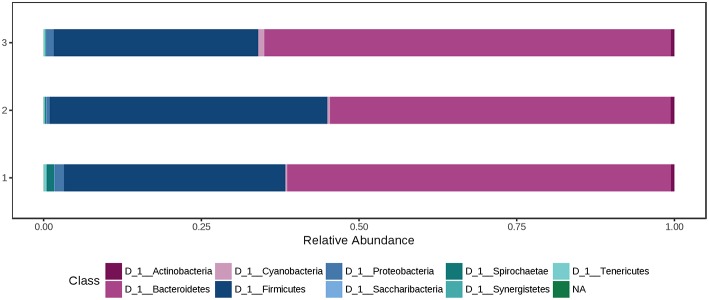
Bacterial relative abundance at phylum level from cecal content depending on the feeding concept (1 = Ctr [0% peat]; 2 = H1.5 [1.5% peat], and 3 = H3.0 [3.0% peat].

At phylum level, the cecal microbiota was dominated by *Bacteroidetes* (60.0%) and *Firmicutes* (37.0%), followed by *Proteobacteria* (1.08%). Using the Mann-Whitney/Kruskal-Wallis test for univariate statistical comparisons between the diet groups, significant differences were found for bacterial relative abundance for *Tenericutes* at phylum level and *Mollicutes* at class level (*p* < 0.05; Supplementary Table 1). The relative abundance of *Firmicutes* and *Bacteroidetes* in the cecal contents did not differ between groups (Ctr: 35.1% *Firmicutes*, 60.8% *Bacteroidetes*; H1.5: 44.1% *Firmicutes*; 35.1% *Bacteroidetes*; H3.0: 32.4% *Firmicutes*; 64.5% *Bacteroidetes*; Supplementary Table 1, Figure 4 and Data Sheet 2.

**Figure 5:**
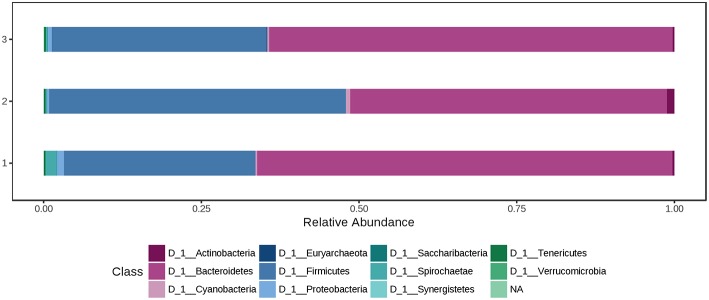
Bacterial relative abundance at phylum level from colon content depending on the feeding concept (1 = Ctr [0% peat]; Class 2 = H1.5 [1.5% peat], and Class 3 = H3.0 [3.0% peat].

At phylum level, the colon microbiota was dominated by Bacteroidetes (60.0%) and Firmicutes (37.0%), followed by Spirochaetae (0.850%) and Proteobacteria (0.726%). Using the Mann-Whitney/Kruskal-Wallis test for univariate statistical comparisons between the diet groups, no significant differences were found for bacterial relative abundance at phylum and class level. The relative abundance of *Firmicutes* in the colon content after offering a 1.5% peat diet was 47.0%, whereas in the other two groups percentages were 30.3% (Ctr) and 34.1% (H3.0). *Bacteroidetes* had a relative abundance of 65.8% (Ctr), 50.2% (H1.5), and 64.0% (H3.0) in the colon content of pigs offered the named diets (Supplementary Table 2, Figure 5 and Data Sheet 2).

## Discussion

The study was planned as a first pilot experiment on pigs, testing peat-containing diets in piglet rearing.

### Performance Parameters

Strictly speaking, the study does not allow any conclusions to be drawn concerning the potential impact of peat use on performance. The sample size was too small. This is the greatest limitation with regard to interpreting data. The overall performance was very good. On average, the animals had daily weight gains of 638 g, the animals with the low peat concentration in the diet (H1.5) even having weight gains of 692 g. In recent studies by Goodarzi Boroojeni et al. (24), Danbred × Piétrain genetics piglets aged 25–66 days showed an average body weight gain of 420 g/day with an FCR of 1.46. The diet in that study contained more protein (as-fed per kg, d 25–38: 214 g CP; d 39–66: 201 g CP) but was energetically comparable to the present one (as-fed per kg, d 25–38: 13.6 MJ; d 39–66: 13.3 MJ). In a further study by Walk et al. (25), in a 20-day feeding trial (start 28 ± 3 days) with crossbred pigs (PIC 337) and complete diets (as-fed per kg: 218 g CP), the animals showed an ADWG of 363 g with an FCR of 1.149. This trial was shorter than the present one, so that the data are not completely comparable. Compared to the cited studies, the performance data in the present study were considerably higher. In general, moderate use of peat seems to have no negative impact on performance. Further investigations are therefore necessary to confirm this hypothesis and specifically investigate the effects of peat application on the mode of feed intake and the digestibility of the nutrients under *ad libitum* feeding conditions.

### Intestinal Chyme

Due to the experimental design, it was not intended to test the digestibility of the different diets. As an indicator, the nitrogen content was measured at two locations in the large intestine in order to gain an impression of the concentrations. The control group showed a higher nitrogen content in the cecal chyme. However, the group did not have the highest feed intake. This was highest in the H1.5 group, where the nitrogen and protein contents were lowest. Also, the concentrations of branched-chain SCFA, which indicates protein fermentation in the large intestine, were rather low. As the carbohydrate sources (starch and other fermentable carbohydrates) are exhausted by fermentation in the large intestine, the carbohydrate:N of the cecum decreases and fermentation becomes more and more proteolytic (26). Therefore, parts of the intestinal SCFA may originate from polypeptides which appear to be the main source of the mainly branched-chain SCFA (isobutyrate, valerate, and isovalerate) formed by the metabolism of branched-chain amino acids such as valine, leucine, and isoleucine (27). In humans, protein fermentation could potentially account for about 17% of SCFA found in cecum, and 38% of the SCFA produced in the sigmoid/rectum (27). In a Canadian study with weaned piglets (~40–42 life days at necropsy) (28), the concentrations of isobutyrate, isovalerate, and valerate in the cecal content were higher in animals fed a low Ca and P (5.4 g Ca, 4.5 g P as-fed) control diet (in mmol/kg wet weight; isobutyrate: 3.3; isovalerate: 3.6; valerate: 6.8) as well as in animals with a high Ca and P (10 g Ca, 8.0 g P as-fed) diet (in mmol/kg wet weight; isobutyrate: 2.7; isovalerate: 2.8; valerate: 5.5) compared to the concentrations in our own study. Also, in the colon content, values were higher in the Canadian study (a low Ca and P diet; in mmol/kg wet weight; isobutyrate: 1.9; isovalerate: 2.7; valerate: 3.4; a high Ca and P diet; isobutyrate: 1.6; isovalerate: 2.2; valerate: 2.0) (28). Apart from the low concentrations of branched-chain fatty acids when using peat diets, there is little evidence of a negative effect on protein utilization when peat is used.

The absolute contents of volatile fatty acids were within the range of literature data. In a Canadian study with weaned piglets (~40–42 life days at necropsy), the total SCFA contents were higher (182 mmol/kg wet weight) in animals on a control diet with low Ca and P contents (5.4 g Ca, 4.5 g P as fed), and lower (160.8 mmol/kg wet weight) in animals with high Ca and P contents (10 g Ca, 8.0 g P as fed) compared to the present study (28). Particularly, the cecal propionate was reduced (*p* < 0.05) by high Ca and P (28). In a well-known study (29), lowest concentrations of SCFA were found in the cecum 2 h after feeding. However, these increased to a value of 212 ± 8 mM after 4 h and then remained high for the remainder of the 12-h period (29). The various segments of colon tended to demonstrate their lowest concentrations of SCFA 4 h after feeding. The concentrations of these acids were about 140–160 mM (29). Significantly higher concentrations of SCFA could also be measured in fattening boars when fed *ad libitum* (30). At 218.8 mmol/kg cecal content, the sum of acetate, propionate, and butyrate was considerably higher than in the piglets in the present study. In general, it was noticeable that the use of peat in complete diets generally resulted in lower SCFA contents, in particular the concentrations of acetic and propionic acid and thus the total SCFA concentration in the present study. For ruminants, possible effects of humic acid on fermentation have already been the subject of investigations (17, 18, 31). Humic acid could be used to modulate the ruminal fermentation pattern by shifting ruminal fermentability to more efficient end products (17). In another study, including humic substances had no effect (*p* ≥ 0.19) on DM disappearance, pH or the concentrations of SCFA (31).

In general, the cumulative gas production, which measures the kinetics of fermentation, can also be used to assess microbial population activity (32). This method involves measuring accumulating gas during fermentation in order to obtain a picture of the kinetics of microbial activity of the population acting as a whole. At the end of the fermentation period, samples are taken in order to measure SCFA and NH_3_, as well as substrate utilization (32). This method can be used for digestion of diets with cecal microflora (26). Drawing conclusions from the results of the batch fermentation are not possible at this time. Batch fermentation was limited in its capacity due to the sample volume. Compared to the control, the cumulative gas formation was numerically reduced by 6.33% (H1.5) and 16.5% (H3.0) in 4 h when using peat. The results of our own investigations thus provide only slight indications that peat probably has an effect on microflora as a lower gas formation did occur.

The microbiome analyses in cecal content showed significantly higher values for one of the specific alpha diversity indices. The diversity seemed to be higher when there was no peat in the diet. Similarly, Högberg et al. (33) and Castillo et al. (34) also found a reduction in the diversity index when coarse wheat bran was incorporated in the diet, suggesting an adaptation of the gut microbiota to fibrous diets. Tajima et al. (35) found a reduction in bacterial diversity in the cecum digesta when an antibiotic was added to the diet after weaning. In this context, how the performance is positively affected by antibiotics remains unclear. Nevertheless, possible mechanisms may include a reduction in total bacterial load, suppression of pathogens or increased nutrient absorption by the host or bacterial community remodeling in favor of non-antagonistic or beneficial bacteria and functions (5), to name just a few hypotheses. In the present study, the microbiome analysis provides only slight indications of a substantial shift of microflora when peat is used. These differences seem to be much smaller than the effects on fermentation in general. Therefore, it seems to be less the composition of the flora than the amount or activity of the flora that is altered by the peat.

In the present study, no significant differences could be shown in the relative abundances of the frequently represented species at phylum and class level. Therefore, no conclusions can be drawn yet. In ruminant studies with humic substances, the microbial community structure was largely unaffected (*p* > 0.05) (18). In a Rusitec Study, humic substances decreased the relative abundance of *Proteobacteria* (*p* = 0.04) and increased the relative abundance of *Synergistetes* (*p* = 0.01), and *Euryarchaeota* (*p* = 0.04) (31). Thus, it may make sense to think further about the effects of peat in animal nutrition.

## Conclusion

The results of this pilot study do not contradict the previous findings from studies on the effects of peat in animal nutrition. In the future, further studies under conditions of high performance in weaning pigs on the effects of the digestibility of feed and the interactions between peat, intestinal microflora, health, and performance should be investigated.

## Author Contributions

GB: conceived the idea for this study; GB and CV: designed the study; CV, JH, AN, BK, EG, TS, CK, and GB: performed the study and the analyses; CV and JH: performed the statistics and wrote the manuscript. All authors read and approved the final version of this manuscript.

### Conflict of Interest Statement

The authors declare that the research was conducted in the absence of any commercial or financial relationships that could be construed as a potential conflict of interest.
