# Supplementary material for: Performance, Fermentation Characteristics and Composition of the Microbiome in the Digest of Piglets Kept on a Feed With Humic Acid-Rich Peat
Source: Front Vet Sci. 2019 Feb 12;6:29. doi: 10.3389/fvets.2019.00029 (PMC6380164; doi:10.3389/fvets.2019.00029)
Supplement: Supplementary file 1 [file Data_Sheet_1.docx]

Supplementary Material

Performance, fermentation characteristics and composition of the microbiome in digesta of piglets kept on a feed with humic acid-rich peat

Christian Visscher^1†*^, Julia Hankel^1†^, Andrea Nies^1^, Birgit Keller^1^, Eric Galvez^2^, Till Strowig^2^, Christoph Keller^3^, Gerhard Breves^4^

*** Correspondence:** Corresponding Author: christian.visscher@tiho-hannover.de

# Microbiome Analyses

**Supplementary Table 1** Comparison of relative abundance of cecal microbiome (%) between the diets at the taxonomic levels phylum and class

| Taxa |  | Ctr | H1.5^1^ | H3.0^2^ | P-values | FDR^3^ |
| --- | --- | --- | --- | --- | --- | --- |
| Phylum | Actinobacteria | 0.547 | 0.618 | 0.579 | 0.39455 | 0.66426 |
|  | Bacteroidetes | 60.81 | 53.98 | 64.45 | 0.37908 | 0.66426 |
|  | Cyanobacteria | 0.298 | 0.415 | 0.952 | 0.88692 | 0.91393 |
|  | Firmicutes | 35.14 | 44.06 | 32.44 | 0.37908 | 0.66426 |
|  | Proteobacteria | 1.497 | 0.513 | 1.215 | 0.40252 | 0.66426 |
|  | Saccharibacteria | 0.068 | 0.081 | 0.024 | 0.85183 | 0.91393 |
|  | Spirochaetae | 1.191 | 0.161 | 0.067 | 0.91393 | 0.91393 |
|  | Synergistetes | 0.010 | 0.005 | 0.014 | 0.44284 | 0.66426 |
|  | Tenericutes | 0.418 | 0.136 | 0.242 | 0.038006 | 0.34206 |
| Class | Actinobacteria | 0.427 | 0.417 | 0.526 | 0.39455 | 0.67291 |
|  | Alphaproteobacteria | 0.265 | 0.099 | 0.034 | 0.15107 | 0.59083 |
|  | Bacilli | 2.715 | 7.420 | 6.763 | 0.59452 | 0.77745 |
|  | Bacteroidia | 60.79 | 53.94 | 62.32 | 0.37908 | 0.67291 |
|  | Betaproteobacteria | 0.066 | 0.155 | 0.138 | 0.17377 | 0.59083 |
|  | Chloroplast | 0.030 | 0.053 | 0.023 | 0.37908 | 0.67291 |
|  | Clostridia | 19.08 | 24.19 | 17.20 | 0.40252 | 0.67291 |
|  | Coriobacteriia | 0.115 | 0.189 | 0.050 | 0.17377 | 0.59083 |
|  | Deltaproteobacteria | 0.012 | 0.005 | 0.052 | 0.475 | 0.67291 |
|  | Erysipelotrichia | 0.577 | 0.795 | 0.581 | 0.45384 | 0.67291 |
|  | Gammaproteobacteria | 1.153 | 0.254 | 0.990 | 0.69073 | 0.83875 |
|  | Melainabacteria | 0.268 | 0.362 | 0.929 | 0.85214 | 0.9054 |
|  | Mollicutes | 0.417 | 0.136 | 0.241 | 0.038006 | 0.59083 |
|  | Negativicutes | 12.73 | 11.64 | 7.892 | 0.14515 | 0.59083 |
|  | Spirochaetes | 1.190 | 0.160 | 0.067 | 0.91393 | 0.91393 |
|  | Synergistia | 0.010 | 0.005 | 0.014 | 0.44284 | 0.67291 |

Ctr [0% peat], H1.5 [1.5% peat] and H3.0 [3.0% peat];
^1^ H1.5 was a mixture of 50% Ctr and 50% H3.0 diet
^2^ The humic acid analysis was carried out only in the H3.0 diet; this diet contained 1.06% extractable humic acids
^3^ Discrete False-Discovery Rate

**Supplementary Table 2** Comparison of relative abundance of colon microbiome (%) between the diets at the taxonomic levels phylum and class

| Taxa |  | Ctr | H1.5^1^ | H3.0^2^ | P-values | FDR^3^ |
| --- | --- | --- | --- | --- | --- | --- |
| Phylum | Actinobacteria | 0.306 | 1.182 | 0.275 | 0.10228 | 0.6684 |
|  | Bacteroidetes | 65.84 | 50.22 | 63.97 | 0.75578 | 0.95187 |
|  | Cyanobacteria | 0.269 | 0.684 | 0.303 | 0.8781 | 0.9659 |
|  | Euryarchaeota | 0.010 | 0.007 | 0.108 | 0.66735 | 0.95187 |
|  | Firmicutes | 30.34 | 47.04 | 34.05 | 0.51171 | 0.95187 |
|  | Proteobacteria | 1.108 | 0.334 | 0.651 | 0.52729 | 0.95187 |
|  | Saccharibacteria | 0.045 | 0.030 | 0.032 | 0.96797 | 0.96797 |
|  | Spirochaetae | 1.760 | 0.206 | 0.229 | 0.53259 | 0.95187 |
|  | Synergistetes | 0.014 | 0.002 | 0.012 | 0.16193 | 0.6684 |
|  | Tenericutes | 0.261 | 0.260 | 0.293 | 0.7788 | 0.95187 |
|  | Verrucomicrobia | 0.005 | 0.007 | 0.011 | 0.18229 | 0.6684 |
| Class | Actinobacteria | 0.196 | 0.774 | 0.218 | 0.1367 | 0.84916 |
|  | Alphaproteobacteria | 0.694 | 0.059 | 0.041 | 0.2972 | 0.84916 |
|  | Bacilli | 1.531 | 4.040 | 3.561 | 0.27527 | 0.84916 |
|  | Bacteroidia | 65.79 | 50.18 | 61.39 | 0.82696 | 0.95883 |
|  | Betaproteobacteria | 0.090 | 0.052 | 0.112 | 0.44486 | 0.88972 |
|  | Chloroplast | 0.044 | 0.040 | 0.011 | 0.67706 | 0.95883 |
|  | Clostridia | 15.83 | 26.37 | 16.50 | 0.28938 | 0.84916 |
|  | Coriobacteriia | 0.111 | 0.407 | 0.058 | 0.07502 | 0.84916 |
|  | Deltaproteobacteria | 0.035 | 0.026 | 0.131 | 0.91089 | 0.95883 |
|  | Epsilonproteobacteria | 0.016 | 0.071 | 0.065 | 0.3999 | 0.88972 |
|  | Erysipelotrichia | 0.646 | 1.069 | 1.051 | 0.40252 | 0.88972 |
|  | Gammaproteobacteria | 0.273 | 0.126 | 0.302 | 0.61878 | 0.95883 |
|  | Melainabacteria | 0.226 | 0.644 | 0.292 | 0.8781 | 0.95883 |
|  | Mollicutes | 0.261 | 0.260 | 0.293 | 0.7788 | 0.95883 |
|  | Negativicutes | 12.34 | 15.55 | 12.93 | 0.88692 | 0.95883 |
|  | Spirochaetes | 1.760 | 0.206 | 0.229 | 0.53259 | 0.95883 |
|  | Synergistia | 0.014 | 0.002 | 0.012 | 0.16193 | 0.84916 |
|  | Thermoplasmata | 0.010 | 0.007 | 0.108 | 0.66735 | 0.95883 |
|  | Unknown_Class | 0.045 | 0.030 | 0.032 | 0.96797 | 0.96797 |

Ctr [0% peat], H1.5 [1.5% peat] and H3.0 [3.0% peat];
^1^ H1.5 was a mixture of 50% Ctr and 50% H3.0 diet
^2^ The humic acid analysis was carried out only in the H3.0 diet; this diet contained 1.06% extractable humic acids
^3^ Discrete False-Discovery Rate
